# Targeted destruction of HIV-positive cells

**DOI:** 10.7448/IAS.17.4.19707

**Published:** 2014-11-02

**Authors:** Jyoti R Sharma, Cleo Dodgen, Amanda Skepu, Mervin Meyer

**Affiliations:** 1 Biotechnology, University of Western Cape, Bellville, Cape Town, South Africa; 2 Nanotechnology Innovation Centre, Mintek, Randburg, South Africa

## Abstract

**Introduction:**

HIV/AIDS is now a global epidemic that has become the leading infectious killer of adults worldwide. Although antiretroviral (ARV) therapy has dramatically improved the quality of life and increased the life expectancy of those infected with HIV but frequency of dosing and drug toxicity as well as the development of viral resistance pose additional limitations. The rapidly expanding field of nanotechnology has vast potential to radically advance the treatment and prevention of HIV/AIDS. Nanoparticles can provide improved drug delivery, by virtue of their small size, robustness, safety, multimodality or multifunctionality.

**Aims and objectives:**

Since HIV primarily infects CD4+ cells; we aim to use CD4 as a selectable target to deliver a pro-apoptotic protein to HIV-infected cells using nanoparticles as carriers. The aim of study was to develop a nanotechnology-based death inducing delivery system for the destruction of CD4+HIV infected cells through the activation of caspase-3.

**Methodology:**

A modified caspase-3 protein (Mut-3) was engineered, which is cleavable only by HIV-1 protease. Mut-3 can activate apoptosis in the presence of HIV-1 protease, consequently killing HIV-positive cells. Mut-3 protein was conjugated to gold nanoparticles together with a CD4-targeting peptide. The efficacy of the gold nanoparticles was tested on CHO cells that were genetically engineered to express GFP labelled CD4 and HIV-1 protease.

**Results:**

Mut-3 was expressed in bacterial cells and purified. CHO cells that stably over express CD4-GFP and HIV-1 protease were selected using Fluorescence Activated Cell Sorting. Dose response cell culture experiments showed that gold nanoparticles without Mut-3 and CD4-targeting peptide did not induce cell death in CHO cells, while gold nanoparticles that was conjugated with Mut-3 and the CD4-targeting peptide rapidly induced cell death in CHO cells.

**Conclusions:**

Our results suggest that gold nanoparticles conjugated with Mut-3 and a CD4-targeting peptide could potentially induce apoptosis in HIV-infected cells.

